# The chromosomes of the Filariae

**DOI:** 10.1186/1475-2883-4-10

**Published:** 2005-11-02

**Authors:** Rory Post

**Affiliations:** 1 Department of Entomology, The Natural History Museum, Cromwell Road, London SW7 5BD, UK; 2 Department of Infectious and Tropical Diseases, London School of Hygiene and Tropical Medicine, Keppel Street, London WC1E 7HT, UK

## Abstract

An understanding of the nature of the chromosomes of the filariae is expected to greatly assist the future interpretation of genome data. Filarial development is not eutelic, and there does not seem to be a fixed number of cell divisions in the way that there is in *Caenorhabditis*. It is not clear whether the chromosomes of the filariae have localized centromeres or whether they are holocentric. Sex determination is by a chromosomal "balance" X0 system in most filariae, but in some Onchocercidae there has been a chromosomal fusion to create a neo-XY system. It is presumed that the molecular basis of sex determination in filariae is similar to *Caenorhabditis*. The ancestral karyotype of the filariae is probably 5A+X0, but in some Onchocercidae this has been reduced to 4A+XY, and in *O. volvulus *and *O. gibsoni *it has been further reduced to 3A+XY. *Onchocerca volvulus *and *O. gibsoni *both have supernumary (B-) chromosomes and in *O. volvulus *there is a single active nucleolus organising region near the middle of the long autosome.

## Background

Filariae and other nematodes have small genomes relative to other multicellular eukaryotes. *Onchocerca volvulus *and *Wuchereria bancrofti *have estimated haploid genomes of 1.5 and 0.81 × 10^8 ^nucleotide pairs respectively [1], and this corresponds, for example, to genome sizes of 30 and 2.78 × 10^8 ^in humans and *Anopheles gambiae *respectively [2]. Because of the small genome size, the chromosomes of the filariae are correspondingly small. This makes it difficult to see their gross morphological features, which are at the limits of resolution of the light microscope, and consequently the cytogenetics of the Filariae have not been well studied. However, the physical structure of the chromosomes is a reflection of the organisation of the DNA and its genetics. For example, the chromosomes determine the number of linkage groups and the pattern of sex linkage, and how this can vary between species. The interpretation of the available nuclear genome sequence of *Brugia malayi *and the expressed sequence tag (EST) libraries for *Onchocerca volvulus*, *Onchocerca ochengi*, *Wuchereria bancrofti*, *Brugia malayi*, *Dirofilaria immitis *and *Litomosoides sigmodontis *[3] as well as any possible *O. volvulus *genome sequencing project will be helped by an understanding of their chromosomes.

## Eutely, centromeres and sex determination

The observations of Goldschmidt [4] established the idea that postembryonic growth in nematodes occurred without further cell division, and this became known as eutely. For more than 70 years this idea became established in the general literature (for example, [5]), but it is now clear that there are many species where it is not true. For example in *Caenorhabditis elegans *there is a 1.47-fold increase in the number of somatic nucleii [6] and in *Romanomermis culicivorax *there is an 8-fold increase during the parasitic phase [7]. However, these two species contrast another feature of nematode development. In *C. elegans *it is clear that there is a fixed number of cell divisions (including post-embryonic cell divisions) during development leading to a constant number of somatic nucleii in the adult [6]. This is not true of *R. culicivorax *where the number of somatic nucleii varies and is correlated with adult size [7]. There have been no specific studies of these patterns in Filariae, but Bain [8] has reported somatic cell divisions in developing larvae of *O. volvulus *in the vector. Furthermore, counts of lacto-acetic orcein stained nucleii [9] of intrauterine microfilariae and infective L3 larvae of *O. volvulus *have indicated a mean of 280 nucleii in microfilariae and approximately 900 in L3s [10]. It is clear that the concept of eutely can not be applied to filariae, because there is obviously an increase in cell number between these two post-embryonic stages. It is also unlikely that filariae have a fixed number of cell divisions because larvae at the same stage of development were found to show variation in numbers of somatic nuclei [9].

It seems that most nematodes probably have holocentric chromosomes (the chromosomes attach to the meiotic and mitotic spindle microtubules along their whole length, instead of this function being concentrated into a single centromere) [11]. A consequence of this is that broken fragments of chromosomes can still assort regularly at cell division. However, it is clear that the trichurids (at least) have normal (localised) centromeres, but the situation is not well understood for the filariae. Procunier and Hirai [12] interpreted their mitotic and meiotic metaphases from *O. volvulus *and *O. gutturosa *in terms of localised centromeres, but it is not obvious that their drawn figures are correct interpretations of their photographs, and they explained that "the position of the centromere is not always obvious" and in the longest chromosome its position "appears to vary between individuals". The chromosomes of the filariae are very small, difficult to interpret and no other authors have directly addressed this issue. However, the orientation of the chromosomes at metaphase has been remarked upon. Delves et al. [13] showed that synaptonemal complexes were present in early female meiosis of *Dirofilaria immitis*, but at metaphase I the chromosomes appeared to be pairing end to end. This was particularly obvious for the X chromosome pair (which is the longest chromosome in *D. immitis*) because a more normal side by side pairing was observed in only 10% of ova. This same end to end pairing is apparent in the photographs of other authors for other filariae (for example [14]). In most animals meiotic metaphase chromosomes show evidence of crossing over (which is the visible manifestation of recombination), but this is not obvious for filariae. Procunier and Hirai [12] interpreted their figures to show crossing over, but no other authors have done so, and it is not clear whether this is due to the small size of the chromosomes or some more fundamental biological reason. In any case, recombination is expected to occur in filariae because they have synaptonemal complexes [13] and recombination has been proven in some other nematodes such as *Caenorhabditis *[15], and *Globodera *[16].

Some nematodes such as *Strongyloides *exhibit forms of parthenogenesis, and a few such as *Mermis subnigrescens *have environmental sex determination (dependant upon the number of mermithids parasitising a particular insect host). However, amongst the vertebrate parasites chromosomal sex determination seems to be the rule. And there seems to be an X0 system in all species where it is known except the Oxyuridae (which have a system of haplo-diploidy), Ascaridae (which have multiple sex chromosomes) and Filarioidea (where some species of Onchocercidae have an XY system). It is clear that the X0 system is fundamental to nematodes (including the filariae), and the few XY systems which occur amongst the filariae are secondary derivatives. In other organisms with X0 sex chromosomes, such as Orthoptera, the fusion of an autosome to the X chromosome to create a neo-XY is very well documented [17]. Genetically, an X0 sex determining system has to be a 'balance' system, and there is a good understanding of the molecular basis of sex determination in *Caenorhabditis *(which has an X0 system, with X0 males and XX hermaphrodites) [18], and it is probable that filariae are basically similar.

## Chromosome numbers and karyotype evolution

**Figure 1:**
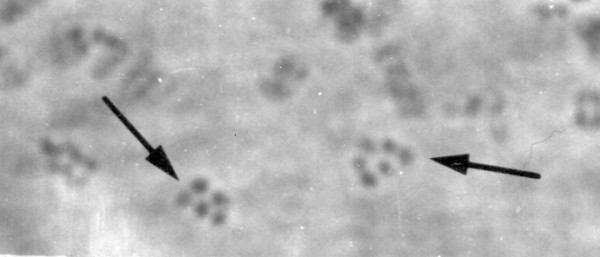
*Litomosoides sigmodontis *karyotype. Aceto-orcein stained spermatozoa from the seminal receptacle of an adult female worm, showing five or six condensed chromosomes (arrowed).

Table 1 summarises all published records of filarial karyotypes, and there are apparently three basic types, 5A+X0, 4A+XY and 3A+XY. There are a few exceptions in the literature, and it is not always clear the extent to which these might be errors resulting from the difficult nature of filarial chromosomes, or indicative of natural variation. *Litomosoides sigmodontis *has been reported to have either 5A+X0 (new unpublished data, see Figure 1) or 4A+X0 [19,20]. In view of the karyotypes of other X0 species it is likely that 5A+X0 is correct, and 4A+X0 is either the result of natural variation for an autosome-autosome translocation or a mistake (due to small difficult chromosomes). Indeed, there are clearly six chromosomes present in some of McLaren's [20] illustrations. Similarly Taylor [19] reported *Dirofilaria immitis *to have 4A+X0, whilst other authors reported 5A+X0 [20] or 4A+XY [21,13]. It is possible that this reflects natural variation within *Dirofilaria*, but Taylor [19] did not have access to a past body of published work that later cytogeneticists have been able to build upon, and it seems most likely that 4A+X0 was a mistake. Post et al. [22] reported that *Onchocerca tarsicola *from Germany was 4A+XY (i.e. n = 5), but the same species from Sweden was n = 3. This difference was attributed to possible intraspecific geographic variation. Most authors agree that *O. volvulus *has 3A+XY (i.e. n = 4). However, Basáñez et al. [23] reported n = 4 or 5, and Miller [24] reported n = 5. It is likely that these reports are the result of B-chromosome variation (see below), but this can not explain the old report by Salazar Mallen et al. [25] of n = 2. In the absence of corroborating evidence this report might be thought of as an error resulting from the experimental use of tissue sections, which is an older cytogenetic technique that is more problematic than the newer squash techniques [26].

**Table 1:** List of karyotypes recorded from the Filarioidea

**Species**	**Karyotype**	**Authors**
**With X0 System:**		
*Acanthoceilonema viteae*	5A+X0	[34]
	5A+X0	[20]
*Dipetalonema setariosum*	5A+X0	[20]
*Setaria equina*	5A+X0	[35]
*Setaria digitata*	5A+X0	[21]
*Litomosoides sigmodontis*	4A+X0	[19]
	4A+X0	[20]
	5A+X0	Jolley & Post, unpublished
*Litomosoides galizai*	5A+X0	Wade & Post, unpublished
*Mononema martini*	5A+X0	Wade & Post, unpublished
*Loa loa*	5A+X0	[36]
**With XY System:**		
*Dirofilaria immitis*	4A+X0	[19]
	5A+X0	[20]
	4A+XY	[21]
	4A+XY	[13]
*Brugia pahangi*	4A+XY	[37]
*Brugia malayi*	4A+XY	[37]
	4A+XY	[38]
*Wuchereria bancrofti*	4A+XY	[24]
*Onchocerca gutturosa*	4A+XY	[39]
	4A+XY	[38]
	4A+XY	[14]
*Onchocerca lienalis*	4A+XY	[14]
*Onchocerca armilatta*	4A+XY	[14]
*Onchocerca tarsicola*	Germany 4A+XY (n = 5)	[22]
	Sweden n = 3	[22]
*Onchocerca dukei*	4A+XY	[22]
*Onchocerca ochengi*	4A+XY	[14]
*Onchocerca gibsoni*	3A+XY	[14]
*Onchocerca volvulus*	n = 2	[25]
	n = 4 or 5	[23]
	n = 5	[24]
	3A+XY (n = 4)	[39]
	3A+XY	[12]
	3A+XY	[40]
	3A+XY	[41]
	3A+XY	[14]

**Figure 2:**
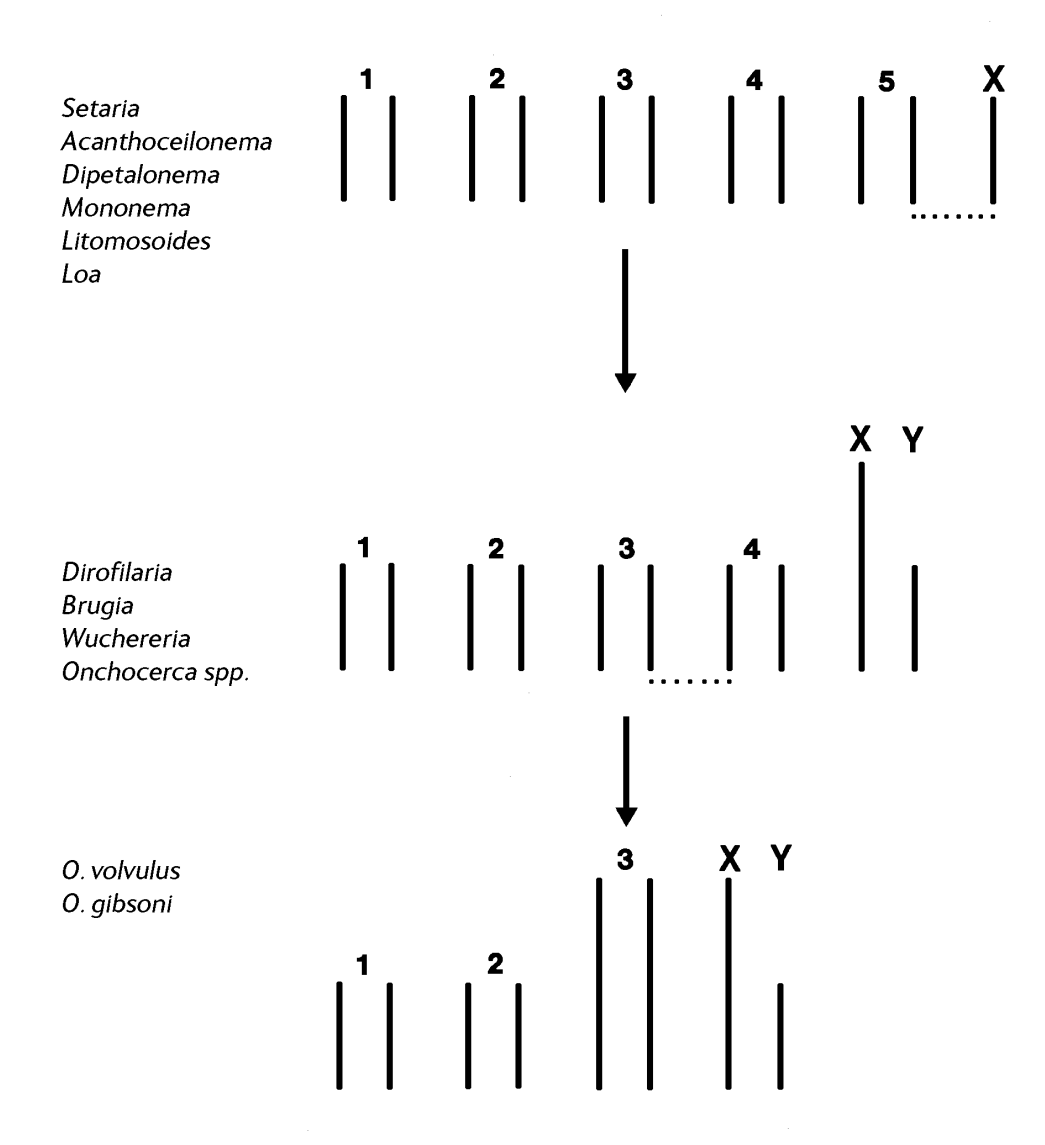
Karyotype evolution in the Filariae.

Figure 2 shows the likely karyotype evolution in the filariae. 5A+X0 is likely to be ancestral because the X0 sex chromosome system is the almost universal sex chromosome system amongst nematodes (see above), and hence the XY system is almost certainly a derived (neo-XY) system, resulting from the fusion of an autosome to the old X-chromosome. There are a number of chromosomal mechanisms for such a fusion [27]. If the chromosomes have localized centromeres a Robertsonian translocation is most likely (i.e. one that occurs at the centromere so that the resultant fragments still have a single centromere and hence can disjoin regularly at meiosis and mitosis). If the chromosomes are holocentric almost any sort of fusion can occur, because all fragments will show centromeric activity and can disjoin regularly. In support of the postulated fusion (by whatever chromosomal mechanism), the five autosomes and the X-chromosome of the 5A+X0 species are all approximately the same size as each other, whereas in the 4A+XY species the four autosomes and the Y-chromosome are of comparable size, whereas the X-chromosome (which would be an X-autosome fusion product) is visibly larger. The 3A+XY karyotype observed in *O. volvulus *and *O. gibsoni *is likely to have been derived from the 4A+XY karyotype by a fusion between two autosomes [12]. The relative sizes of the different chromosomes are consistent with this hypothesis in that one of the autosomes is clearly the largest chromosome, and presumably the autosome-autosome fusion product.

The pattern of karyotype evolution (Figure 2) is likely to reflect organismal evolution and indicate taxonomic relationships. All species of filarioids which have been examined for their chromosomes are from the family Onchocercidae, and from three subfamilies, Setariinae (*Seteria *spp), Dirofilariinae (*Dirofilaria *and *Loa*) and Onchocercinae (all other species in Table 1). The presumed ancestral state (5A+X0) occurs in the Setariinae, most of the Onchocercinae and *Loa loa *(but not *Dirofilaria*). Current phylogenetic opinion has been unable to resolve the major clades of the Onchocercinae, which reduce to a star polytomy [28]. *Loa *forms a clade from this unresolved polytomy, but *Dirofilaria *is consistently grouped along the *Onchocerca *clade. The reduction to 4A+XY has occurred in both *Dirofilaria *and *Onchocerca*, and hence it supports the molecular data, but it also indicates that the *Onchocerca*/*Dirofilaria *clade is most closely related to the *Wuchereria*/*Brugia *clade because it is found in both.

The observation that *O. volvulus *apparently shares the same karyotype as *O. gibsoni*, but not *O. ochengi *or *O. dukei*, might be taken as evidence for a close phylogenetic relationship, and Muller [29] also considered that *O. volvulus *was most closely related to *O. gibsoni *on the basis of the structure of the cuticle. However, Bain [30] held that *O. volvulus *was taxonomically more closely related to *O. ochengi*, and recent molecular phylogeny reconstruction using the 12s, 16s and ND5 mitochondrial genes very strongly supports this view (Morales Hojas, Cheke and Post, unpublished data). If this is true, it would indicate that the similar karyotypes of *O. volvulus *and *O. gibsoni *have been produced by two different fusion events (which may not even have involved the same pairs of autosomes), and this is likely to be reflected in the precise position of the translocation breakpoints at the molecular level.

## B-chromosomes and nucleolus organising region

Both *O. volvulus *and *O. gibsoni *have B-chromosomes [12,14]. These are supernumery chromosomes which are not present in every individual, and when they are present they can be haploid, diploid, triploid, tetraploid, etc, in different individuals. The origin and subsequent evolution of B-chromosomes is a long-standing problem in genetics [31]. B-chromosomes can have definite phenotypic affects, but it has always been unclear whether this was the result of specific genes or a mere consequence of the presence of an extra chromosome. It is largely unknown whether B-chromosomes carry genes and whether those genes are repeated elsewhere in the more constant part of the genome. In any case, if the origin of the 3A+XY karyotype was the result of some sort of autosome-autosome fusion this would result in one new large autosome (see Figure 2), and possibly a small chromosomal fragment [27]. The exact nature of the fragment will depend upon the exact nature of the fusion and there are a number of potential chromosomal mechanisms, which partly depend upon the exact nature of the chromosomes. Procunier and Hirai [12] interpreted the B-chromosomes of *O. volvulus *as chromosomal fragments which resulted from a Robertsonian translocation (i.e. a translocation at the site of the centromere in a species with localised centromeres) which resulted in a new large autosome and a small chromosomal fragment. In such cases, both products would be able to assort regularly because both would have a centromere. If *Onchocerca *have holocentric chromosomes, any chromosome fragment will show centromeric activity and disjoin regularly.

**Figure 3:**
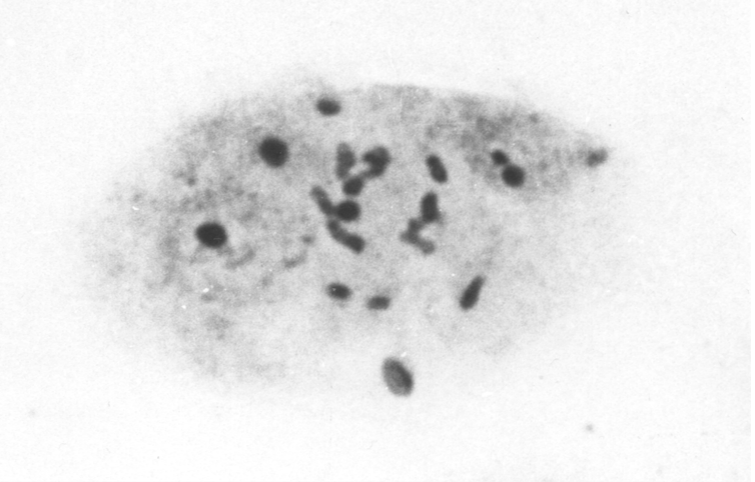
*Onchocerca volvulus *NOR. Silver stained seven-cell morula embryo showing six interphase nuclei and the central cell in mitotic metaphase with four chromosome pairs including the longest pair each with a central Nucleolus Organising Region darkly stained.

There have been no attempts to characterize the different chromosomes of any filariae using chromosome banding or molecular cytogenetics. The only differences that have been noted are size differences and sex chromosome differences (see above). However, There has been one unpublished study of silver staining of *O. volvulus *(Post & Bella, unpublished data). Silver staining is a technique that stains the nucleolus and hence reveals the active nucleolus organising regions, which is where the active ribosomal genes (rDNA) are found [32]. In *O. volvulus *there was a single nucleolus organizing region, situated at the centre of the long autosome (Figure 3). There may be other inactive clusters of rDNA elsewhere in the genome, and it is interesting to note that rDNA has sometimes been implicated at the sites of chromosome mutations (such as translocations) in other organisms such as humans [33].

## Conclusion

1. Eutely is certainly not a characteristic of filarial development, and there does not seem to be a constant number of somatic cells at different developmental stages. However, it is still possible that the rather constant pattern of cell lineages seen in *Caenorhabditis *is present in filariae in some more flexible form.

2. Although some authors have interpreted their metaphase chromosome figures as indicating localised centromeres this is unusual in nematodes, which usually have holocentric chromosomes. Meiotic metaphase chromosomes exhibit an unusual appearance of end to end pairing in filariae, which is not consistent with having a localised centromere. The nature of the centromere in filariae remains to be resolved.

3. Sex determination in filariae is chromosomal and fundamentally of the X0 type, although in some species of the Onchocercidae this has been secondarily modified into a neo-XY system by the fusion of an autosome onto the old X chromosome. X0 sex determination is genetically a balance system, and likely to be similar to that described at the molecular level in *Caenorhabditis*.

4. The primitive karyotype of the Filarioidea is presumed to be 5A+X0, because the X0 chromosomal sex determining system seems to be almost universal amongst nematodes in general. However, in some Onchocercidae there has been an X-autosome fusion to produce a neo-XY system with four autosomal pairs (4A+XY). In *O. volvulus *and *O. gibsoni *there has been a further fusion of two autosomes to yield a karyotype of 3A+XY. This second step might have occurred independently in the two species.

5. Both *O. volvulus *and *O. gibsoni *have B-chromosomes, probably resulting from the autosome-autosome translocation which reduced the karyotype to n = 4. However, nothing is known about these chromosomes in terms of their genetics or potential phenotypic effects.

6. In *O. volvulus *there is a single active nucleolus organizing region (where the active rDNA occurs) near the centre of the long autosome, near the site of the chromosomal fusion event.

## Competing interests

The author(s) declare that they have no competing interests.
